# Nanoparticles boost pomegranate growth and defense, suppressing root-knot nematodes

**DOI:** 10.3389/fpls.2025.1560126

**Published:** 2025-08-04

**Authors:** Dalia A. Abdel-Wahab, Aida M.I. El-Zawahry, Afaf M. Hamada, Maha M. Abdel-Salam, Ahmed M. Samy

**Affiliations:** ^1^ Botany Department, Faculty of Science, New Valley University, El Kharja, Egypt; ^2^ Plant Pathology Department, Faculty of Agriculture, Assiut University, Assiut, Egypt; ^3^ Botany and Microbiology Department, Faculty of Science, Assiut University, Assiut, Egypt; ^4^ Pomology Department, Faculty of Agriculture, Assiut University, Assiut, Egypt; ^5^ Department of Plant Pathology, Higher Institute for Agricultural Cooperation and Extension, Assiut, Egypt

**Keywords:** CuO nanoparticles, Fe_2_O_3_ nanoparticles, *Meloidogyne javanica*, pomegranate, photosynthetic pigments, non-enzymatic antioxidants

## Abstract

Root-knot nematodes (*Meloidogyne* spp.) are a major threat to pomegranate cultivation. Nanoparticles (NPs) present a possible substitute nematicide that lessens dependency on potentially dangerous chemical nematicides. This study assessed the efficacy of copper oxide (CuO) and iron oxide (Fe_2_O_3_) NPs to promote pomegranate (*Punica granatum* L. cultivar Hegazy) growth and provide protection against the root-knot nematode (*Meloidogyne javanica*). The application of the NPs as copper oxide (CuO) and iron oxide (Fe_2_O_3_) involved both drenching and spraying using 50 mg/L on one-year-old pomegranate (*Punica granatum* cultivar Hegazy) seedlings, nematode-infected with (*Meloidogyne javanica*). By assessing how CuO and Fe_2_O_3_ NPs affect nematode and pomegranate growth, and some biochemical traits. Treatments with NPs successfully reduced the number of pomegranate root egg masses, galls, and juvenile nematodes in soil. NP treatments exhibited increased side branching, leaf area, levels of photosynthetic pigments (chlorophyll *a*, *b*, and carotenoids), total antioxidants, thiol compounds [glutathione (GSH), non-protein thiols (NPTs), protein thiols (PTs)], and flavonoids. However, NP treatments reduced the accumulation of malondialdehyde (MDA) and proline, stress markers, in pomegranate plants infected with nematodes. NP treatments did not affect the production of phenolic compounds in pomegranates. These results indicate that the NP effect partially depends on the increased production of photosynthetic pigments, thiol compounds, and flavonoids. These results elucidate how nanoparticles control nematode infection. Further research in this area is necessary to determine whether NPs are the best treatment for nematode infections.

## 1 Introduction

Pomegranate (*Punica granatum* L.) is an ancient fruit tree regarded as a “Superfood” and has long been used as an herbal remedy (Adhami et al., 2012). Globally, pomegranate cultivation extends across 500,000 hectares, yielding over 6.3 million tons annually. Egypt has 33,816 hectares of pomegranate cultivation, where 672,064 tons were produced in 2020, and this area is growing (Singh et al., 2022; Hussein et al., 2023). Assiut Governorate is widely acknowledged as the primary region for pomegranate cultivation in Egypt (Reclamation M.o.A.a.L, 2012). In Assiut, 10,404 feddans are cultivated with pomegranates, yielding approximately 201589.329 tons (average yield: 17.63 tons/feddan).

The severity of nematode attacks on crops, a significant problem across their global growing areas, is particularly pronounced in arid climates and light sandy soils (Singh et al., 2019). The impact of these nematodes is significant, leading to 30 to 40% reductions in yield and a noticeable degradation in fruit quality (Holland et al., 2009; Singh et al., 2019). While the specific species and population densities of root-knot nematodes in pomegranate orchards remain largely undocumented, Singh et al. (2022) identified *Meloidogyne incognita* and *M. javanica* as the primary species affecting this crop. According to El-Qurashi et al. (2017), the investigation of pomegranate plantations in the Assiut region confirmed the existence of root-knot nematode species (*Meloidogyne* spp.). *Meloidogyne javanica* is the primary nematode threat to Assiut’s pomegranate orchards. Furthermore, in most of the world’s key pomegranate-producing nations, *M. javanica* is currently the most significant species. Root-knot nematode management is not without its challenges. Various approaches are employed to control nematodes on plant hosts, including chemical, regulatory, physical, cultural, and biological treatments, each having benefits and drawbacks (El-Zawahry et al., 2021). To date, nematodes have been managed using nematicides and soil fumigation, posing a threat to sustainable agricultural development, human health, and environmental pollution, which raises concerns about food safety. Therefore, developing sustainable nematode management strategies is crucial to maintaining agricultural productivity and ensuring food safety.

Nanotechnology is currently attracting a lot of interest from a range of areas due to its essential features; recently, it has been used as a new weapon in pest control due to its unique qualities (Tauseef et al., 2021). The application of nanotechnology in agriculture has garnered attention since it presents a realistic possibility for significantly increased agricultural productivity and the ability to reduce waste and costs (Saha and Dutta Gupta, 2018). Additionally, micronutrients have been implicated in the control of plant parasitic nematodes (e Silva et al., 2025).

Copper and iron are micronutrients that play several roles in phytochemical processes (Marschner et al., 2017). Copper formulations show promise as nematicides, as they can also combat harmful fungi and bacteria (Chatterjee et al., 2014; Bramhanwade et al., 2016). Remarkably, Fe-based nanoparticles (NPs) have a lot of potential in the agricultural sector because they are thought to have low toxicity and are biocompatible (Ali et al., 2016; Yousaf et al., 2024). To our knowledge, there hasn’t been a thorough investigation of how copper oxide (CuO) and ferric oxide (Fe_2_O_3_) NPs affect pomegranate non-enzymatic antioxidants.

Currently, no experimental data exist regarding the effects of NPs on *Meloidogyne javanica* stress. In contrast, silver nanoparticles demonstrated an effect on other *Meloidogyne* sp*ecies* (Furmanczyk et al., 2025). We undertook this study to assess how CuO and Fe_2_O_3_ affect nematode and pomegranate growth, photosynthetic pigments, malondialdehyde, and the non-enzymatic antioxidant system. Pomegranate (*Punica granatum*) was chosen for this investigation due to its economic importance and widespread cultivation in the Assiut Governorate, Egypt, a region characterized by the prevalence of *Meloidogyne javanica* in the soil. The results indicate that CuO and Fe_2_O_3_ NPs have the potential to mitigate pomegranate nematode infections. This effect may be linked to pomegranate’s ability to stimulate the production of total antioxidants, flavonoids, thiol compounds, and photosynthetic pigments.

## 2 Materials and methods

This section details the experimental setup and analytical procedures employed to investigate the effects of copper oxide (CuO) and iron oxide (Fe_2_O_3_) nanoparticles (NPs) on the root-knot nematode *Meloidogyne javanica* under greenhouse conditions, as well as their impact on various physiological and biochemical parameters of pomegranate seedlings. The application of the NPs involved both drenching and spraying on one-year-old pomegranate (*Punica granatum* cultivar Hegazy) seedlings. The study included a two-year greenhouse experiment at the Plant Pathology Department of the Faculty of Agriculture at Assiut University.

### 2.1 Effect of copper oxide and iron oxide nanoparticles on *Meloidogyne javanica* under greenhouse conditions

-Source of copper oxide and iron oxide nanoparticles:
-Copper oxide (50%).
-Iron (111) oxide, industrial, nanoArc@20-40nm Aps powder, S. A. 30-60 m2/g Fe2O3.

#### 2.1.1 Greenhouse experiment

This experiment was conducted in the greenhouse of the Plant Pathology Department, Faculty of Agriculture, Assiut University, for two years in the same season. One-year-old pomegranate seedlings were moved individually into clay pots with a diameter of 35 cm and filled with 4 kg of sterile loamy soil. The pots were divided into treatments based on the following criteria:

- Uninfected control (sterilized soil only) Healthy.
- Infected control (soil + nematode).
- CuO NPs 50 mg/L + nematode (drenching) (selected from a preliminary test).
- CuO NPs 50 mg/L + nematode (spraying) (selected from a preliminary test).
- Fe_2_O_3_ NPs 50 mg/L + nematode (drenching) (selected from a preliminary test).
- Fe_2_O_3_ NPs 50 mg/L + nematode (spraying) (selected from a preliminary test).

To shield the soil from NP contact during spraying, plastic sheets were placed over the pots 24 hours before infestation. The plants were thoroughly covered to the point of dripping with the nano-solution (pH 5.7-6.0) using a hand sprayer. Soil drench treatments, consisting of 10 mL nano-solution injections around the roots, were administered 24 hours before infestation. The control groups were treated with distilled water.

Pomegranate seedlings were simultaneously inoculated with 2,000 *Meloidogyne juveniles* (J2s) in 15 mL of water around the root zone and treated with the designated nanoparticles. *M. javanica* was identified molecularly using SCAR primers and morphologically based on the perineal pattern. In a greenhouse, the pots were arranged haphazardly and given light watering. Each treatment was replicated three times. The plants were uprooted after ninety days, and the soil that was stuck to the roots was carefully washed away (100 g/pot). Juvenile nematodes (J2s) were extracted and counted following the method described by Goodey (1957). The roots were immersed in a 1 mg/L erioglaucine solution for 15 minutes in order to color the egg masses blue (Atamian et al., 2012). The number of root galls was quantified.

#### 2.1.2 Growth parameters

After the plants (36) were uprooted, samples of their leaves were taken in order to evaluate growth factors and subsequently identify them using ImageJ software. To calibrate the image analysis program, leaf blades were positioned on A4 paper with a black rectangle measuring 3 x 15 cm (45 cm^2^). A digital camera with a 7.2-megapixel resolution was then used to take pictures of each leaf, and ImageJ software was used to process the images and determine the area. The leaf and camera were 50 cm apart on average. After photographic processing, the leaves were evaluated using the leaf area meter (LI-COR, 1996). The number of side branches per plant was counted.

### 2.2 Physiological and biochemical parameters

The levels of the photosynthetic pigments (carotenoids, chlorophyll *a*, and *b*) in the leaves of pomegranate plants were determined separately using the spectrophotometric method outlined by Lichthenthale (1987). The units of measurement for their concentrations were in milligrams per gram of fresh weight (mg/g FW).

Using plant organs, lipid peroxidation was estimated as membrane damage. The thiobarbituric acid (TBA) test was thus used to assess MDA. Lipid peroxidation was calculated using the extinction coefficient (155 mM^-^¹ cm^-^¹), and the result was expressed in µM/g FW (Rao and Sresty, 2000).

Using the acid-ninhydrin method, the amount of proline in the leaves and roots of pomegranate plants was determined (Bates et al., 1973). A calibration curve was used to calculate the proline concentration, which was then reported in mg/g FW.

The leaf sample was homogenized using 5 mL of a 5% sulfosalicylic acid solution. The homogenate was centrifuged at 12000 rpm for 20 minutes at 4°C. The resulting supernatant was used for GSH and non-protein thiols. The Anderson (1985) method was used to determine reduced glutathione (GSH). The supernatant was mixed with phosphate buffer (pH 7.0), ethylenediaminetetraacetic acid (EDTA), and 5,5’-dithiobis-2-nitrobenzoic acid (DTNB). The absorbance was measured at 412 nm. GSH content, expressed as μM/g FW, was determined using a GSH calibration curve.

The estimation of the non-protein thiol (NPT) content followed the guidelines provided by Israr et al. (2006). The supernatant was transferred to an Eppendorf tube, to which phosphate buffer (pH 7.0), EDTA, and DTNB were added. Following a 10-minute incubation period at room temperature, the mixture’s absorbance at 412 nm was measured. A calibration curve at various cysteine concentrations was used to calculate the NPT content, which was then reported as μM/g FW. According to Nagalakshmi and Prasad (2001), the content of protein thiols (PTs) was determined by deducting NPTs from total protein thiols, and the result was given as μM/g FW.

Total phenolic concentrations were determined by using the Folin-Ciocalteu reagent method (Sampietro et al., 2009). The methanolic extract was mixed with an equal volume of trichloroacetic acid (TCA) to precipitate the proteins and to eliminate tyrosine, tryptophan, and other nonphenolic reactive compounds (Hatch et al., 1993). The sample was mixed with a freshly prepared solution that included 1% CuSO_4_, 2.7% sodium and potassium tartrate, and 2% Na_2_CO_3_. After 10 minutes of room temperature incubation with 0.5 N NaOH, a diluted Folin reagent was added to the mixture. Spectrophotometric measurement of the absorbance at 750 nm was used to determine the total phenolic content, which was expressed as mg/g DW.

The total flavonoid content was calculated using the Chang et al. (2002) method. The reaction mixture, consisting of the sample extract, 1.2% aluminum chloride, and 120 mM potassium acetate, was incubated for 30 minutes at room temperature. The absorbance was measured at 415 nm. Quercetin was used for the calibration curve (Ordonez et al., 2006). The total flavonoid content was expressed in terms of the standard equivalent (mg/g DW).

Total antioxidants (Anti.Ox.) were measured according to Prieto et al. (1999). A reagent, consisting of 0.6 M sulfuric acid, 28 mM phosphate buffer, and 4 mM ammonium molybdate, was mixed with the methanolic extract. This solution was then incubated in a water bath at 90°C for 90 minutes. The absorbance was measured at 750 nm. Ascorbic acid was used as a standard, and the total antioxidant content was expressed as µg/g DW.

### 2.3 Statistical analysis

Data represent the mean ± SD of three technical replicates and three biological replicates. Data analysis was performed using SPSS software, version 26. A one-way ANOVA was employed to determine significant differences among treatments with or without nematodes. Tukey’s HSD test (*P* ≤ 0.05) was subsequently used for pairwise comparisons. To assess the effect size of each component and the interaction between treatments and nematodes, eta-squared (η^2^) was calculated using the formula η^2^ = SSEffect/SSTotal. All assessed parameters were analyzed using principal component analysis (PCA) and variance regression ordination. The heatmap was generated and visualized using the ggplot and corrplot packages in RStudio. Statistical significance between treatments with or without nematodes was determined using Excel’s t-test (*P ≤ 0.05; **P ≤ 0.01; ***P ≤ 0.001; ns = non-significant).

## 3 Results

This study investigated the potential of CuO and Fe_2_O_3_ NPs, applied via soil drenching or foliar spraying, to enhance the resistance of pomegranate plants against the root-knot nematode *M. javanica*. Our major findings indicate that both CuO and Fe_2_O_3_ NPs generally mitigated the negative impacts of nematode infection.

### 3.1 Effect of CuO and Fe_2_O_3_ NPs on *Meloidogyne javanica*

**Figure 1:**
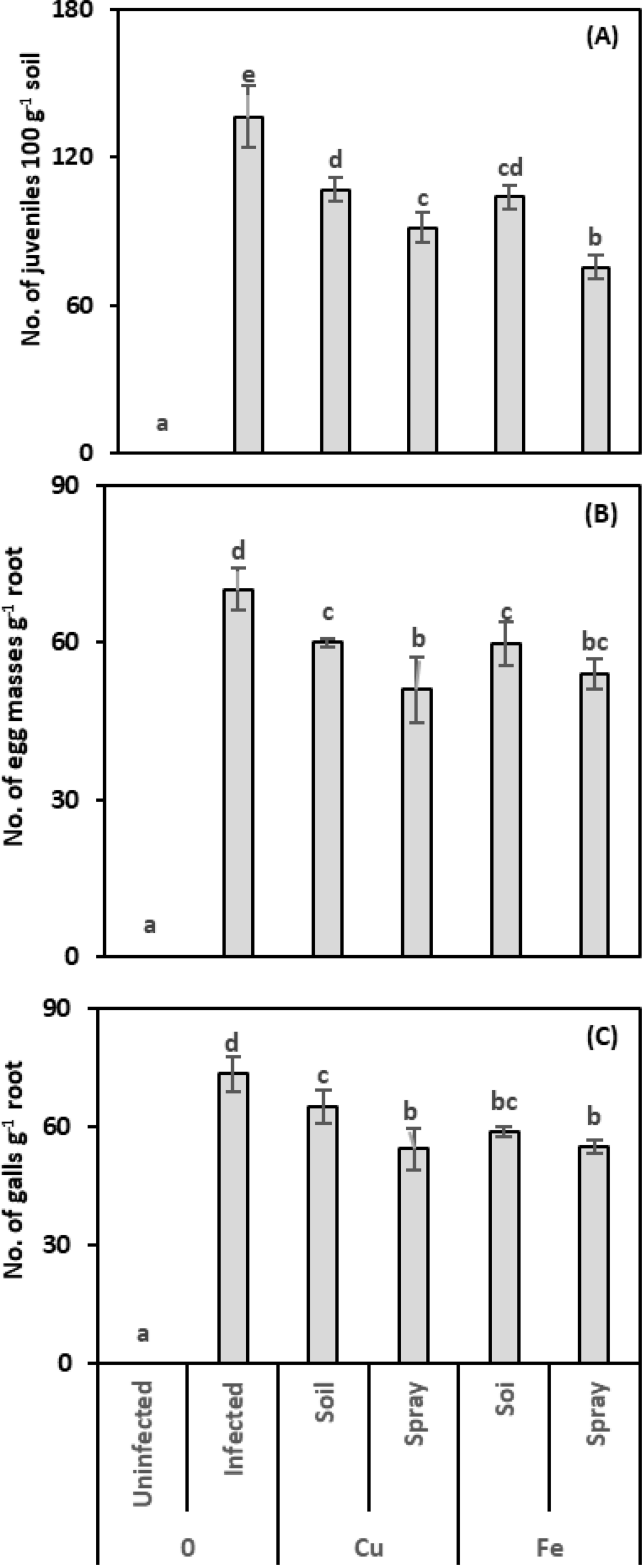
Effect of CuO (Cu) or Fe_2_O_3_ (Fe) nanoparticle treatments (drenching the soil or spraying the plant) on the number of young nematodes per 100 g of soil **(A)**, number of egg masses per one g of root **(B)**, and number of galls per one g of root **(C)** of pomegranate plants under nematode infection. The data are means ± SD (n = 3). The different letters, capital for uninfected and small for infected plants, indicate significance (one-way ANOVA; Tukey HSD *post hoc*).

In this study, we counted the nematode parameters (number of juveniles, egg masses, and root galls) to evaluate the effectiveness of CuO or Fe_2_O_3_ treatments (soil drenching or plant spraying) in enhancing pomegranate plants’ resistance against nematode infection (**Figures 1A-C**). CuO considerably decreased the juvenile population compared to infected plants; declines of 21.61% and 32.97% were observed for drenching and spraying, respectively. Plants treated with Fe_2_O_3_ exhibited fewer juveniles than infected plants alone; equivalent figures for drenching and spraying were 23.81% and 44.69%, respectively. The most significant decrease in the juvenile population was observed upon applying the CuO or Fe_2_O_3_ spray treatment. The Fe_2_O_3_ treatment had the greatest effect on reducing the number of juveniles when administered to nematode-infected plants (η² = 0.641).

Applying CuO to nematode-infected plants reduced the number of egg masses; this reduction was only 14.59% and 27.30% when the soil was drenched and the plant sprayed, respectively. The egg masses for Fe_2_O_3_ treatments in infected plants dropped to 14.95% and 23.13%, respectively, after drenching and spraying, compared to nematode-infected plants. Egg masses fell most when CuO or Fe_2_O_3_ was sprayed on nematode-infected plants; this reduction in egg masses was connected with the number of juveniles. The two-way analysis showed that the application of CuO had the highest size effect on egg masses in the infected plants (η^2^ = 0.577).

CuO and Fe_2_O_3_ treatments reduced the number of root galls. With soil drenching and foliar spraying, CuO reduced root galls by 11.26% and 25.94%, respectively, while Fe_2_O_3_ achieved reductions of 20.14% and 25.26% compared to infected plants. Also, the findings showed that spraying CuO or Fe_2_O_3_ on nematode-infected plants caused the highest decrease in the number of root galls. Two-way analysis showed that Fe_2_O_3_ treatments had the greatest effect on reducing root galls in nematode-infected plants (η² = 0.513).

### 3.2 Effect of CuO and Fe_2_O_3_ NPs on growth parameters of pomegranate infected with *Meloidogyne javanica*

**Figure 2:**
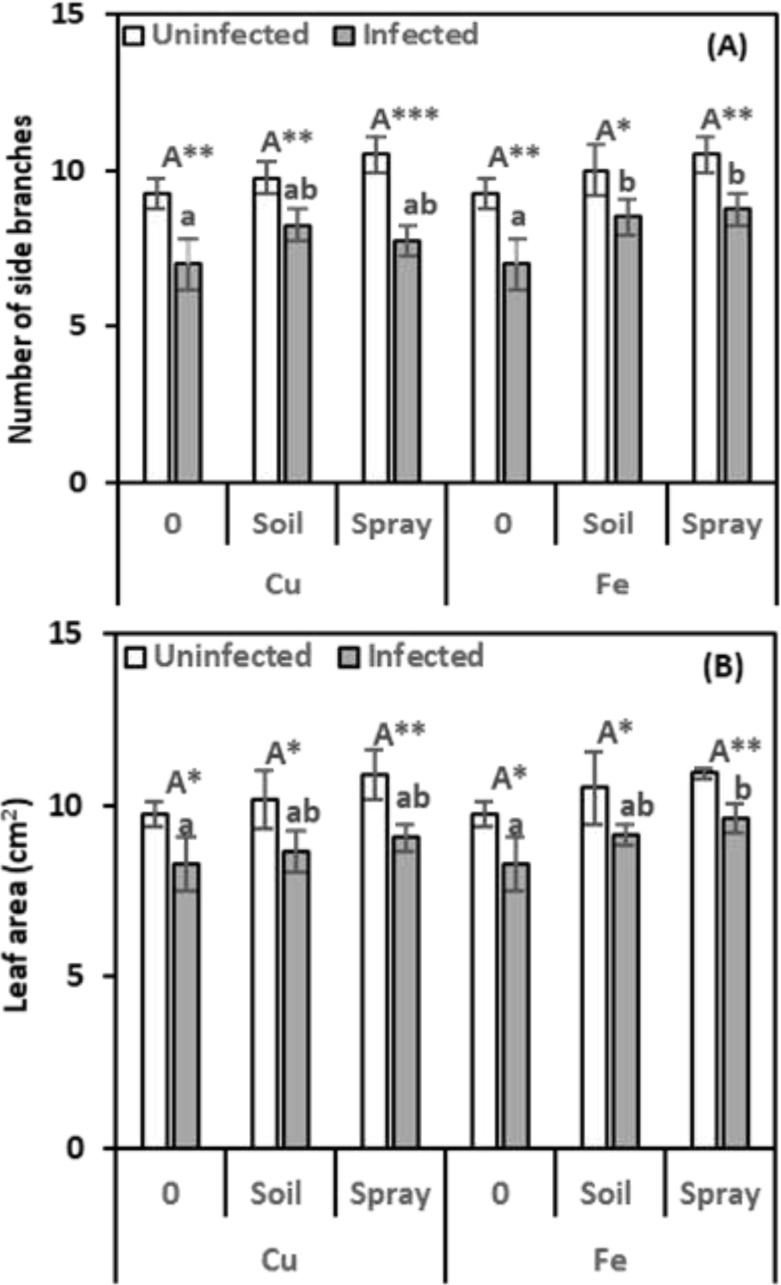
Effect of CuO (Cu) or Fe_2_O_3_ (Fe) nanoparticle treatments (drenching the soil or spraying the plant) on the number of side branches **(A)** and leaf area **(B)** of pomegranate plants under nematode infection. The data are means ± SD (n = 3). The different letters, capital for uninfected and small for infected plants, indicate significance (one-way ANOVA; Tukey HSD *post hoc*), and asterisks indicate significant differences between infected and uninfected plants (t-test; *P ≤ 0.05; **P ≤ 0.01; ***P ≤ 0.001; ns, non-significant).

Nematode infection adversely affected multiple growth indicators in pomegranate plants, including side branch number, leaf area, and photosynthetic pigment levels. In the current investigation, pomegranate seedlings treated with nematode infection exhibited evident toxicity symptoms, reflected in a reduction in growth parameters (**Figures 2A, B**).

Nematode infection significantly reduced plant-side branch development by 24.32% compared to healthy plants (**Figure 1A**). Compared to infected plants, CuO NPs increased side branch growth by 17.86% with soil drenching and 10.71% with foliar spraying. By drenching the soil and spraying the leaves, Fe_2_O_3_ nanoparticles also greatly improved the growth of side branches, increasing them by 121.43% and 125%, respectively.

Compared to healthy plants, CuO or Fe_2_O_3_ treatments alone did not significantly enhance side branch formation with either drenching or spraying. NP treatments significantly influenced side branch development, with Fe_2_O_3_ and infection treatments exhibiting the most pronounced effects (η² = 0.151).

Nematode infection resulted in a 15.10% reduction in the leaf area of infected plants compared to healthy ones. In most cases, CuO applications did not significantly reduce the adverse effects of infection on leaf areas. The increases over infected plants were only 4.36% for soil drenching and 9.48% for foliar spraying treatments. Similarly, when Fe_2_O_3_ was applied to the soil of nematode-infected plants, a non-significant increase in leaf area was observed, with a 10.22% improvement compared to infected plants alone. However, spraying Fe_2_O_3_ on nematode-infected plants resulted in a significant increase in leaf areas, with a 16.00% improvement compared to the infected ones. There was no discernible change in leaf areas after applying CuO or Fe_2_O_3_ alone (**Figure 1B**). CuO and nematode infection were significantly different (η^2^ = 0.148) from Fe_2_O_3_ and nematode infection (η^2^ = 0.128), according to the findings of a two-way ANOVA.

**Figure 3:**
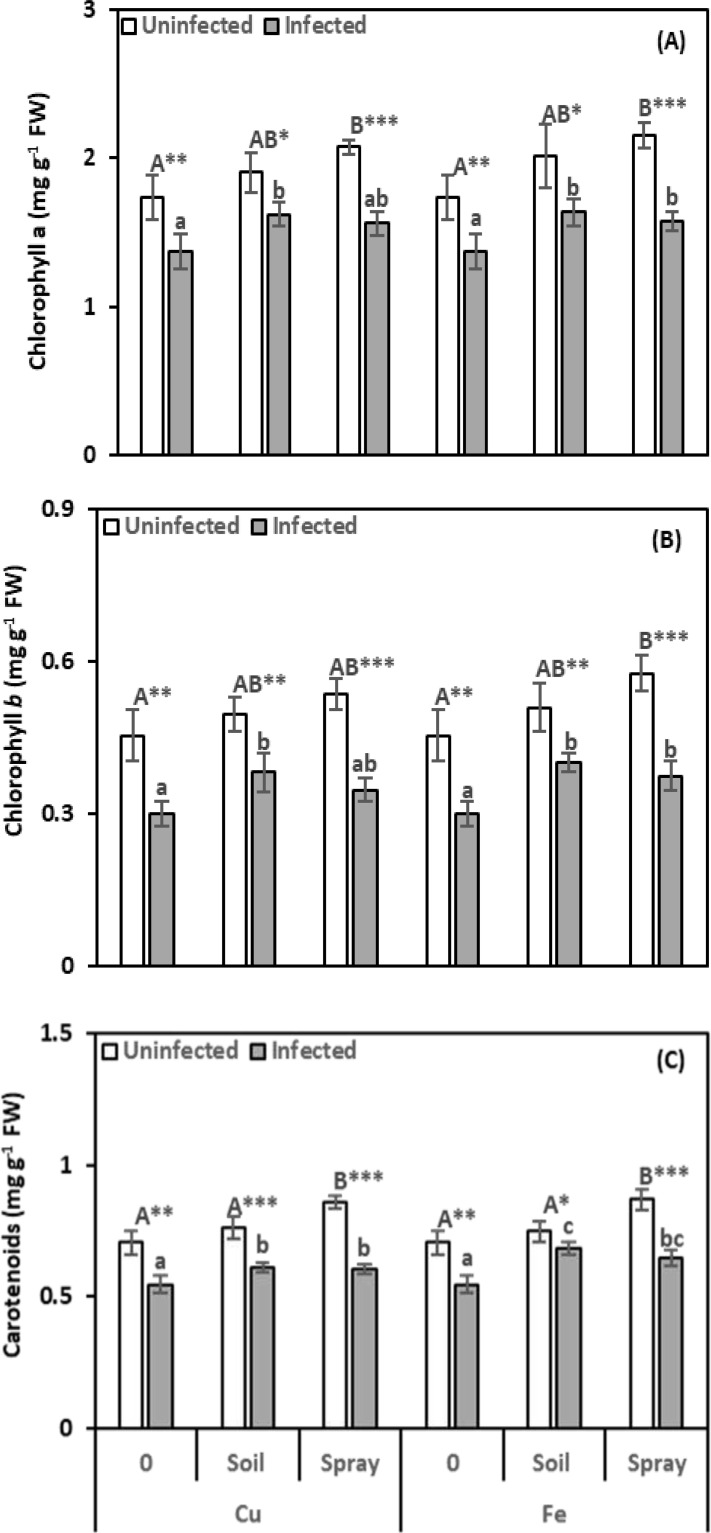
Effect of CuO (Cu) or Fe_2_O_3_ (Fe) nanoparticle treatments (drenching the soil or spraying the plant) on chlorophyll *a* **(A)**, chlorophyll *b* **(B)**, and carotenoids **(C)** of pomegranate plants under nematode infection. The data are means ± SD (n = 3). The different letters, capital for uninfected and small for infected plants, indicate significance (one-way ANOVA; Tukey HSD *post hoc*), and asterisks indicate significant differences between infected and uninfected plants (t-test; *P ≤ 0.05; **P ≤ 0.01; ***P ≤ 0.001; ns = non-significant).

Nematode infection had deleterious effects on photosynthetic pigments (**Figures 3A-C**). Chlorophyll *a*, *b*, and carotenoid levels significantly decreased by 20.74%, 33.91%, and 22.53%, respectively, compared to healthy plants. Applications of CuO or Fe_2_O_3_ increased photosynthetic pigments, helping to reduce the effects of nematode infection. However, chlorophyll *a* and *b* levels did not significantly change in plants sprayed with CuO. Applying CuO or Fe_2_O_3_ alleviated nematode infection by increasing photosynthetic pigments. However, plants sprayed with CuO did not show significant changes in chlorophyll *a* and *b* levels. Compared to infected plants, CuO increased chlorophyll *a*, *b*, and carotenoid levels by 17.94%, 27.43%, and 11.63% with soil drenching and 13.46%, 15.78%, and 10.75% with spraying, respectively. Similarly, Fe_2_O_3_ increased chlorophyll *a*, *b*, and carotenoid levels by 18.92%, 33.44%, and 24.86% with soil drenching and by 14.92%, 24.91%, and 18.08% with spraying, respectively. In most cases, control plants sprayed with CuO or Fe_2_O_3_ alone showed significant increases in photosynthetic pigments, but soil-applied NPs had no appreciable impact on them. The greatest effect on chlorophyll *a* was moderate for CuO and nematode infection (η² = 0.101), whereas Fe_2_O_3_ with nematode infection had a strong impact on chlorophyll *b* and carotenoid levels (η² = 0.144 and 0.157, respectively).

### 3.3 Effect of CuO and Fe_2_O_3_ NPs on stress indicators of pomegranate infected with *Meloidogyne javanica*

**Figure 4:**
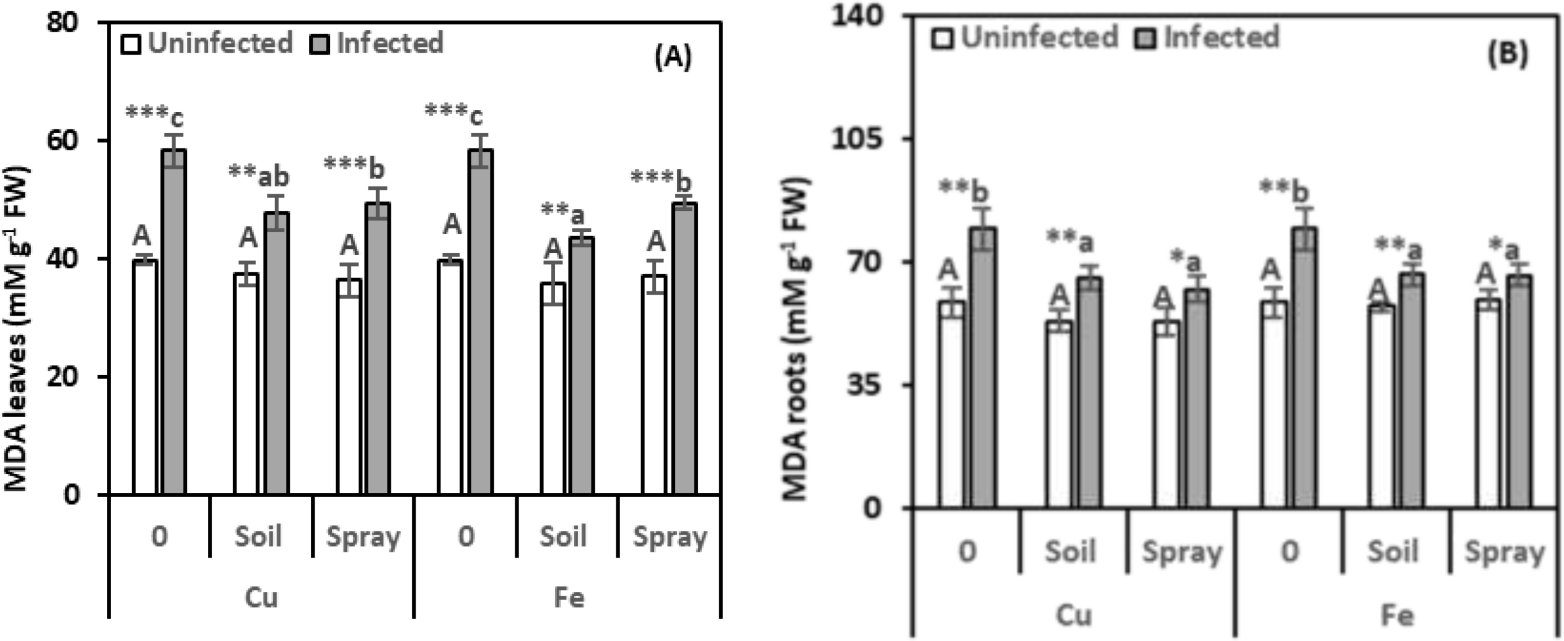
Effect of CuO (Cu) or Fe_2_O_3_ (Fe) nanoparticle treatments (drenching the soil or spraying the plant) on malondialdehyde (MDA) content of leaves **(A)** and roots **(B)** of pomegranate plants under nematode infection. The data are means ± SD (n = 3). The different letters, capital for uninfected and small for infected plants, indicate significance (one-way ANOVA; Tukey HSD *post hoc*), and asterisks indicate significant differences between infected and uninfected plants (t-test; *P ≤ 0.05; **P ≤ 0.01; ***P ≤ 0.001; ns, non-significant).

**Figure 5:**
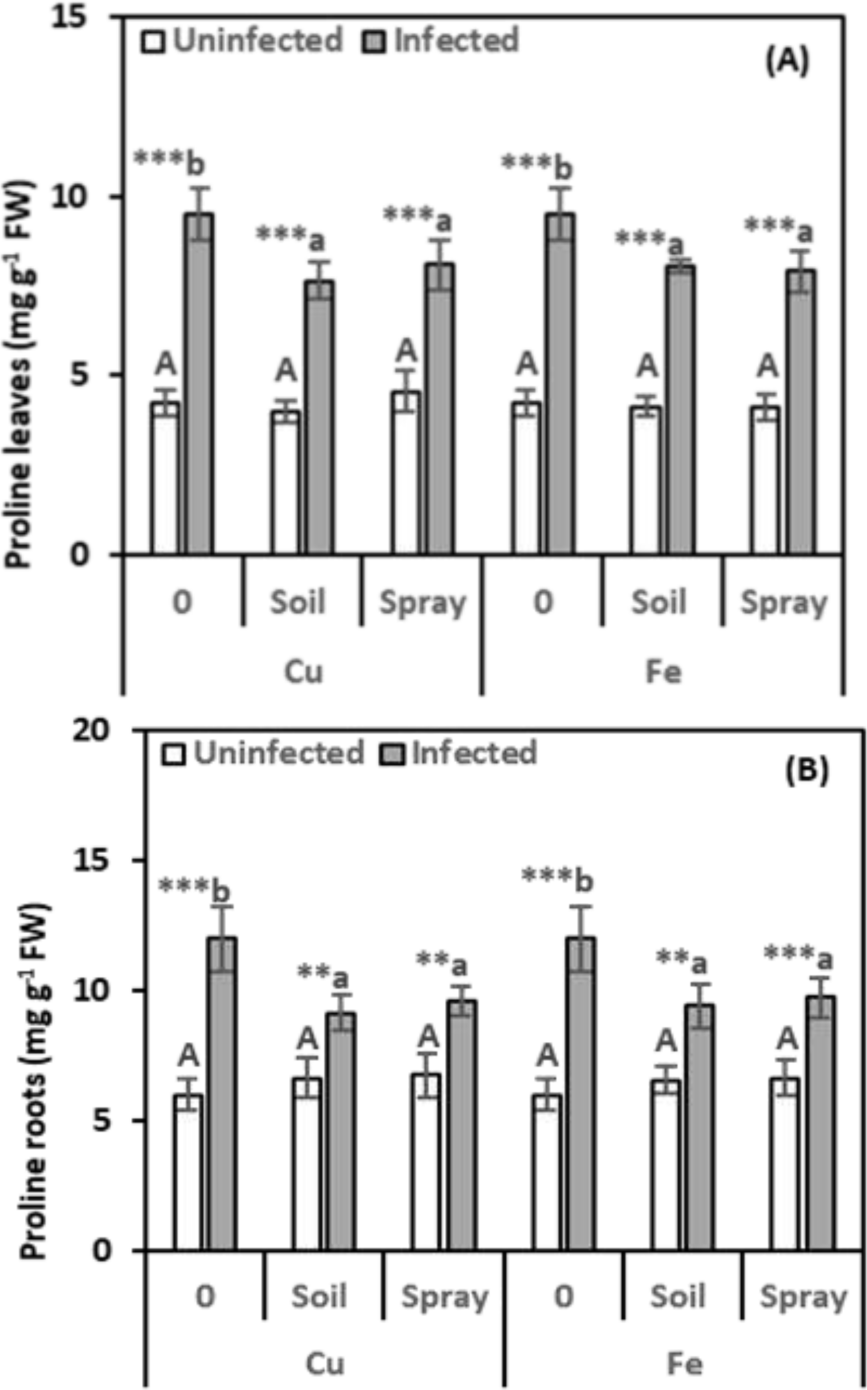
Effect of CuO (Cu) or Fe_2_O_3_ (Fe) nanoparticle treatments (drenching the soil or spraying the plant) on proline content of leaves **(A)** and roots **(B)** of pomegranate plants under nematode infection. The data are means ± SD (n = 3). The different letters, capital for uninfected and small for infected plants, indicate significance (one-way ANOVA; Tukey HSD *post hoc*), and asterisks indicate significant differences between infected and uninfected plants (t-test; *P ≤ 0.05; **P ≤ 0.01; ***P ≤ 0.001; ns, non-significant).

Stress markers, including MDA and proline in pomegranate leaves and roots, were examined to assess the extent of harm caused by nematode infection and the efficacy of NPs in minimizing such adverse effects (**Figures 4**, **5**). The nematode infections raised the MDA concentration in leaves and roots by 46.38% and 35.41%, respectively, compared with healthy plants (**Figures 4A, B**). However, all NP treatments exhibited a recovery effect, reducing MDA levels compared to nematode-infected plants. For instance, CuO treatments applied to the soil reduced MDA content by 17.93% in leaves and 17.34% in roots. Nevertheless, spraying treatments led to reductions of 15.38% in leaves and 21.50% in roots compared to infected plants. Compared to infected plants alone, Fe_2_O_3_ treatments significantly reduced MDA concentrations in nematode-infected plants. Soil drenching applications decreased MDA levels by 25.15% in leaves and 16.36% in roots. Spraying reduced MDA levels by 15.08% in leaves and 16.42% in roots. NPs alone, added to healthy plants, did not significantly alter their MDA content. According to the two-way analysis, CuO treatments significantly affect MDA content in leaves and roots, respectively (η^2^ = 0.201 and 0.252).

Proline levels were significantly higher in infected plants, leaves, and roots, showing 124.84% and 99.84% increases, respectively, over healthy plants (**Figures 5A, B**). Proline levels in infected plants dropped with NP treatments, regardless of the method used, although they remained higher than in healthy plants. CuO treatments significantly lowered proline levels in infected leaves and roots compared to infected plants. Soil drenching with CuO led to 19.54% and 23.61% decreases in proline content in leaves and roots, respectively. Foliar spraying with CuO resulted in 14.85% and 19.91% reductions in proline levels in leaves and roots, respectively. Similarly, Fe_2_O_3_ treatments significantly reduced proline accumulation in infected leaves and roots. Soil drenching and foliar spraying with Fe_2_O_3_ decreased proline levels by 15.40% and 16.84% in leaves and 21.53% and 18.73% in roots, respectively, compared to infected plants. The proline content did not significantly alter when NPs were applied to healthy plants. The results of the two-way analysis revealed that the CuO and nematode treatments had the highest size effect on the proline content in leaves (η^2^ = 0.205), while the Fe_2_O_3_ and nematode treatments had the highest size effect on the roots (η^2^ = 0.276).

### 3.4 Effect of CuO and Fe_2_O_3_ NPs on phytochelatin levels in pomegranate infected with *Meloidogyne javanica*

**Figure 6:**
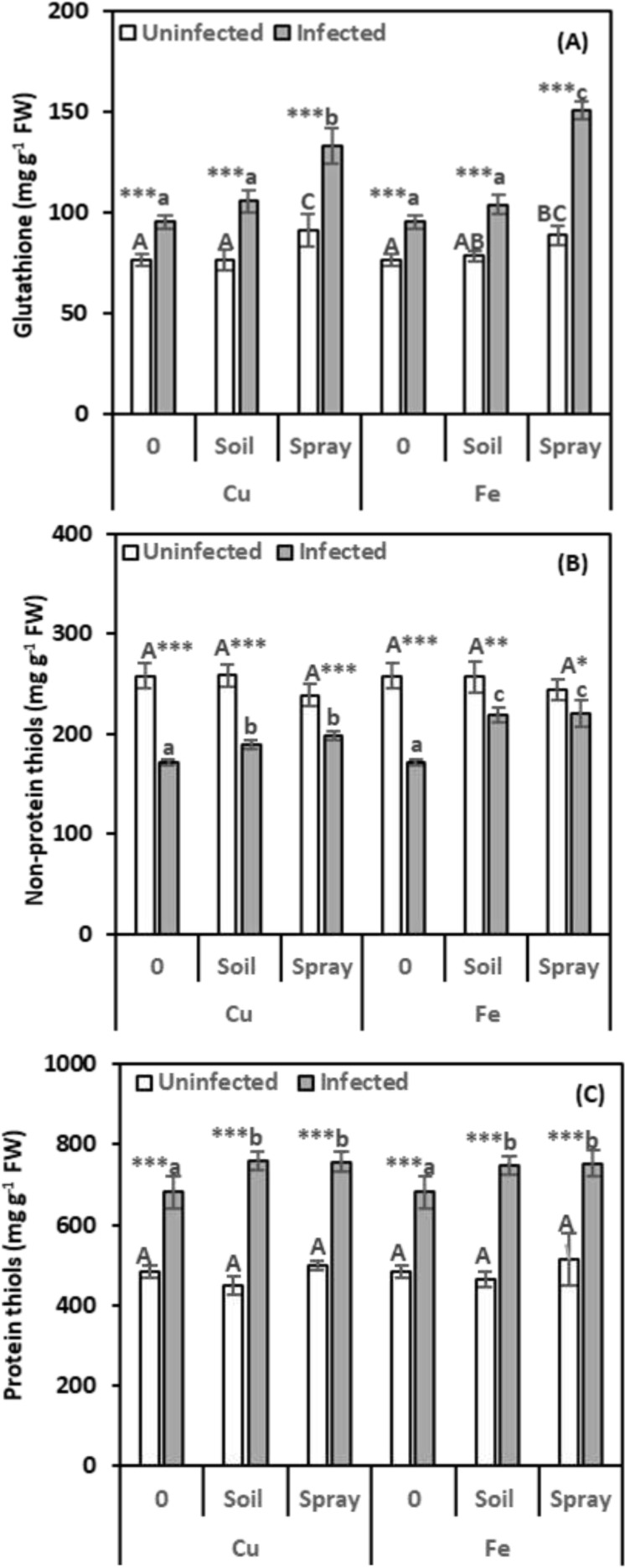
Effect of CuO (Cu) or Fe_2_O_3_ (Fe) nanoparticle treatments (drenching the soil or spraying the plant) on glutathione **(A)**, non-protein thiols **(B)**, and protein thiols **(C)** of pomegranate leaves under nematode infection. The data are means ± SD (n = 3). The different letters, capital for uninfected and small for infected plants, indicate significance (one-way ANOVA; Tukey HSD *post hoc*), and asterisks indicate significant differences between infected and uninfected plants (t-test; *P ≤ 0.05; **P ≤ 0.01; ***P ≤ 0.001; ns = non-significant).

It is essential to measure glutathione (GSH) levels because it influences several physiological functions. Nematode infection boosted GSH levels by 24.89% compared to healthy plants (**Figure 6A**). NP treatments considerably raised the GSH concentration in nematode-infected leaves compared to infected plants alone. The best method for increasing GSH content in nematode-infected leaves was to apply a CuO or Fe_2_O_3_ spray. Compared to infected plants, this technique boosted GSH by 39.99% and 58.44%, respectively. GSH levels considerably rose in healthy plants that received CuO or Fe_2_O_3_ sprays but did not increase significantly when NPs were drenched. Based on the two-way analysis, Fe_2_O_3_ treatments affect the GSH content in nematode-infected plants (η^2^ = 0.064).

Since non-protein thiols (NPTs) are known to protect plants from stress, evaluating them was essential for this investigation. According to **Figure 6B**, nematode infection resulted in a considerable drop in NPTs (33.26%) compared to healthy plants. CuO treatments boosted NPT levels in infected plants. Soil drenching increased levels by 10.08%, and foliar spraying increased them by 15.06% compared to infected plants. Similarly, Fe_2_O_3_ demonstrated significant increases in NPTs of 27.22% and 28.11%, respectively, compared to infected plants alone. Plants treated with NPs alone did not show appreciable modifications in their NPTs. The two-way analysis indicates that CuO treatments affected the NPT content in plants infected with nematodes (η^2^ = 0.256).

Given the significant role of protein thiols (PTs) in detoxification and redox equilibrium, assessing their levels was crucial for this study. The findings in **Figure 6C** demonstrate that nematode infection significantly increased the level of PTs in leaves by 40.64% compared to healthy plants. The combined treatment of NPs and nematodes significantly enhanced PT accumulation in infected plants. CuO and Fe_2_O_3_ treatments significantly enhanced PT accumulation in infected plants. Soil drenching and foliar spraying with CuO or Fe_2_O_3_ increased PT levels by 11.61% and 11.22% (CuO) and 10.07% and 10.61% (Fe_2_O_3_), respectively, compared to infected plants. Plants treated with NPs alone showed no significant differences in PTs from uninfected plants. In nematode-infected plants, CuO had the highest size effect on protein thiols (η^2^ = 0.061).

### 3.5 Effect of CuO and Fe_2_O_3_ NPs on non-enzymatic antioxidant levels in pomegranate plants infected with *Meloidogyne javanica*

**Figure 7:**
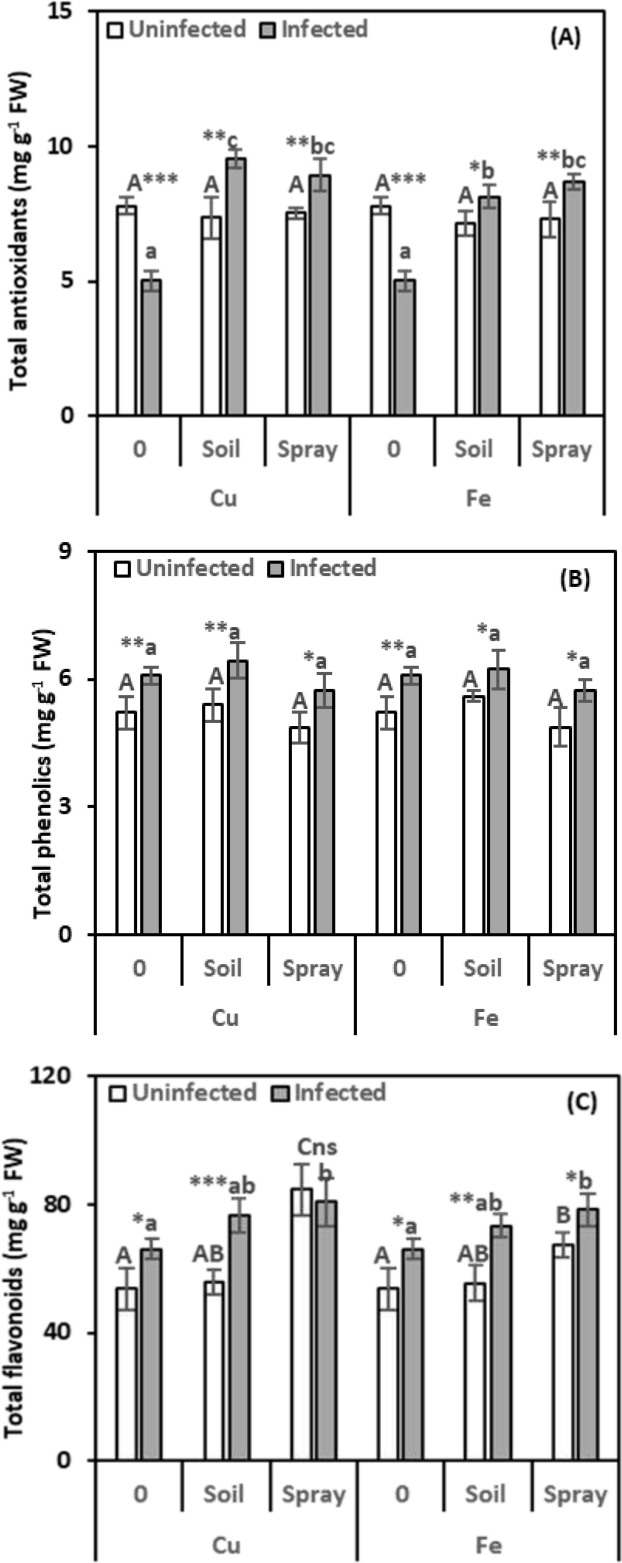
Effect of CuO (Cu) or Fe_2_O_3_ (Fe) nanoparticle treatments (drenching the soil or spraying the plant) on total antioxidants **(A)**, total phenolics **(B)**, and total flavonoids **(C)** of pomegranate leaves under nematode infection. The data are means ± SD (n = 3). The different letters, capital for uninfected and small for infected plants, indicate significance (one-way ANOVA; Tukey HSD *post hoc*), and asterisks indicate significant differences between infected and uninfected plants (t-test; *P ≤ 0.05; **P ≤ 0.01; ***P ≤ 0.001; ns = non-significant).

We measured total antioxidants to see how well the cells can fight oxidative stress (**Figure 7A**). Compared to healthy plants, nematode infection significantly reduced total antioxidant levels in the leaves by 35.60%. However, the antioxidant content of NP-treated nematode-infected plants increased significantly compared to healthy plants. CuO treatments significantly increased the total antioxidant content in nematode-infected plant leaves compared to infected plants. Soil drenching with CuO led to a 90.05% increase in the total antioxidant content, while foliar spraying with CuO resulted in a 77.56% increase. Fe_2_O_3_ treatments also boosted total antioxidant levels in infected plant leaves. Soil drenching increased levels by 62.00%, and foliar spraying increased them by 72.42% compared to infected plants. When NPs were given alone to healthy plants, there was no appreciable increase in the total antioxidant content in the leaves. Fe_2_O_3_ had the highest impact on total antioxidant levels in infected plants (η² = 0.184).

The evaluation of phenolics, secondary metabolites that reduce oxidative stress by warding off harmful molecules known as ROS, was essential. Based on the data presented in **Figure 7B**, it can be noticed that nematode infection had a considerable stimulatory effect on phenolic compound levels (16.63%) in the leaves compared to healthy plants. However, this stimulation was unaffected by NP treatments applied to nematode-infected plants. Uninfected plants treated with NPs showed no discernible change in the level of phenolics. Fe_2_O_3_ had the highest effect on phenolic levels in infected plants (η² = 0.202).

Evaluation of flavonoids, secondary metabolites that can reduce ROS by capturing free radicals and acting as hydrogen donors, was essential. As shown in **Figure 7C**, nematode infection caused a significant increase in leaves’ flavonoid levels by 22.91% over healthy plants. Both nematode infections and NP treatments significantly enhanced the content of flavonoids. Compared to infected plants, flavonoid levels increased by 16.03% and 10.91% following nematode infection and soil drenching with either CuO or Fe_2_O_3_, respectively. CuO or Fe_2_O_3_ sprays also boosted flavonoid levels in infected leaves by 22.01% and 18.37%, respectively. Without nematode infection, spraying plants with NPs boosted leaf flavonoids, but adding them to the soil had no effect. CuO had the highest impact on leaf flavonoids in infected plants (η² = 0.141).

### 3.6 Principal component analysis and hierarchical heatmap clustering pattern

**Figure 8:**
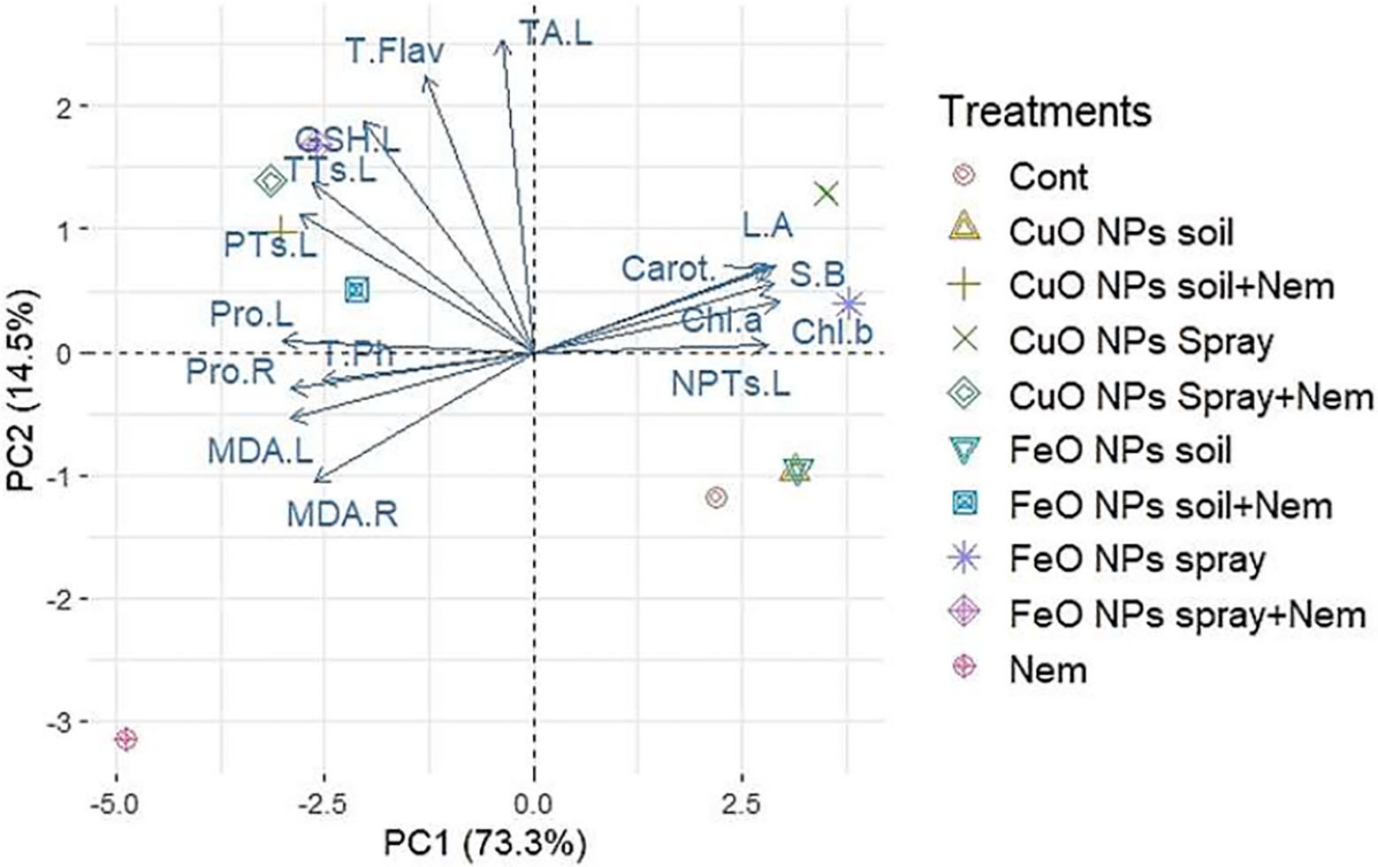
Principal component analysis (PCA) of the studied parameters of pomegranate plants infected with nematodes in combination with applying CuO (Cu) or Fe_2_O_3_ (Fe) nanoparticle treatments (drenching the soil or spraying the plant). Chl *a*, Chlorophyll *a*; Chl *b*, Chlorophyll *b*; Carot, Carotenoids; S.B., side branches; L.R., Leaf area; NPTs.L, non-protein thiols in leaf; TA.L, total antioxidant in leaf; T.Flav, total flavonoids; GSH.L, glutathione in the leaf; TTs.L, total thiols in the leaf; PTs.L, protein thiols in the leaf; T.Ph, total phenolics; Pro.L, proline in the leaf; Pro.R, proline in the root; MDA.L, malonaldehyde in the leaf; MDA.R, malonaldehyde in the root.

To increase the discriminatory power for grouping measured parameters, Principal Component Analysis (PCA) was applied to the experimental dataset. This dataset comprised 16 physiological traits and 9 treatments, which involved either CuO or Fe_2_O_3_ NP applications (via soil amendment or foliar spray) under both control and nematode-infected conditions. Two primary components contributed to 87.8% of the observed variability in the leaf and root data. Specifically, PC1 accounted for 73.3%, while PC2 explained 14.5% (**Figure 8**). The original trait data’s PCA provides a clear description of all pertinent positive and negative correlations between the traits under evaluation. Under nematode infection stress, PCA indicated positive correlations among the non-enzymatic antioxidant [total phenolics (T.Ph.)] and stress indicators [malondialdehyde of leaves (MDA.L), malondialdehyde of roots (MDA.R.), and proline of roots (Pro.R.). Under nematode infection and with the application of CuO (Cu) or Fe_2_O_3_ (Fe) nanoparticle treatments (either soil application or foliar spray), PCA revealed positive correlations among non-enzymatic antioxidants [total flavonoids (T.Flav.) and total antioxidants of leaves (TA.L)] and phytochelatin compounds [glutathione of leaves (GSH.L.), total thiols of leaves (TTs.L.), and protein thiols of leaves (PTs.L.)]. These correlations are visible in the left-hand half of the biplot in **Figure 8**. Conversely, when CuO and Fe_2_O_3_ nanoparticles were applied as a foliar spray, PCA revealed positive correlations among growth traits [side branches (S.B.) and leaf area (L.A.), photosynthetic pigments [chlorophyll a (Chl *a*), chlorophyll b (Chl *b*), and carotenoids (Carot.)], and the phytochelatin compound non-protein thiols of leaves (NPTs.L.). These relationships are displayed in the right-hand half of the biplot in **Figure 8**.

**Figure 9:**
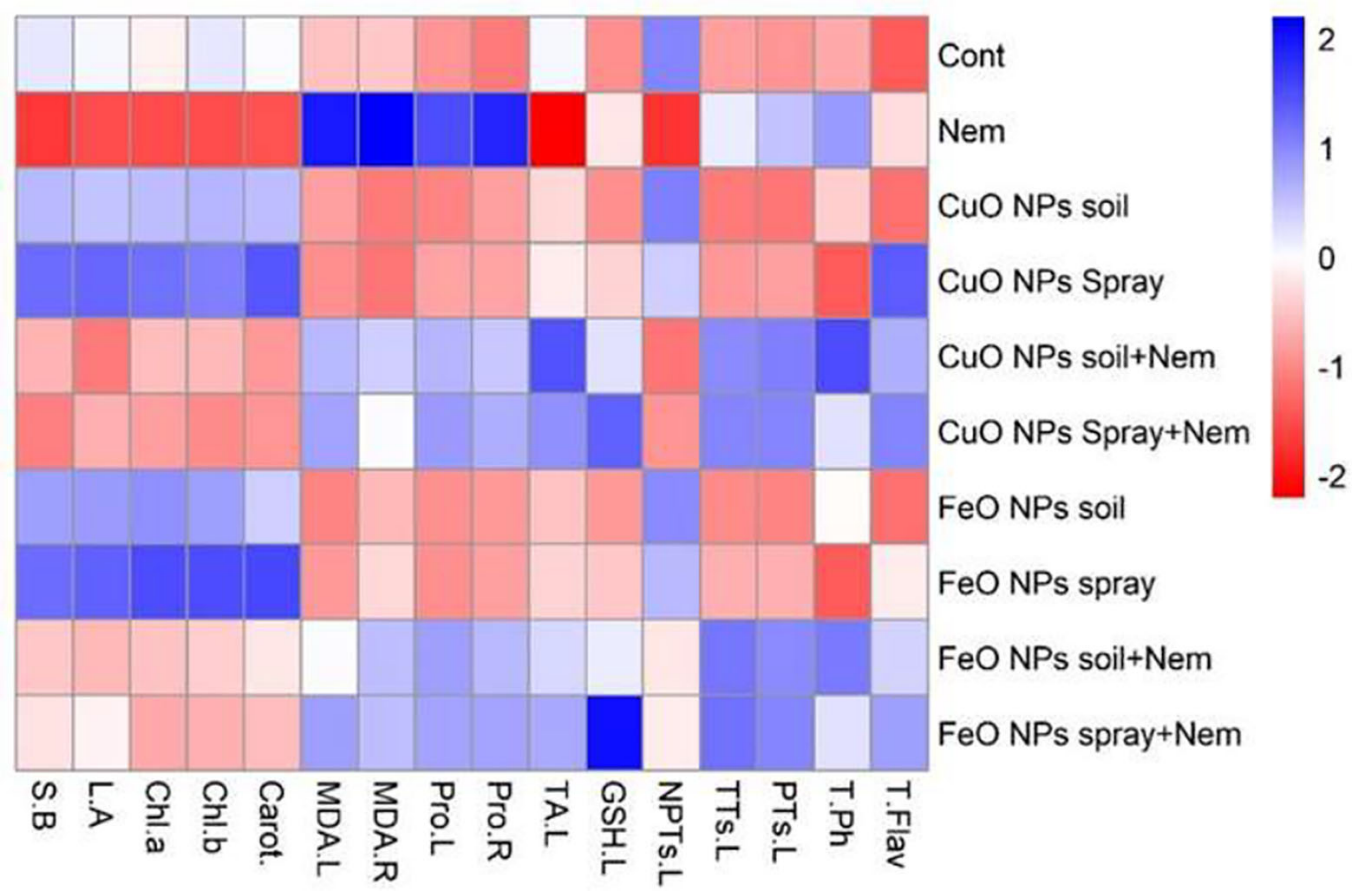
Heatmap showing the saturation of colors indicating pomegranate plants infected with nematode combined with applying CuO (Cu) or Fe_2_O_3_ (Fe) nanoparticle treatments (drenching the soil or spraying the plant). Chl *a*, chlorophyll *a*; Chl *b*, chlorophyll *b*; Carot, carotenoids; S.B, side branches; L.R, Leaf area; NPTs.L, non-protein thiols in leaf; TA.L, total antioxidant in leaf; T.Flav, total flavonoids; GSH.L, glutathione in the leaf; TTs.L, total thiols in the leaf; PTs.L, protein thiols in the leaf; T.Ph, total phenolics; Pro.L, proline in the leaf; Pro. R, proline in the root; MDA.L, malonaldehyde in the leaf; MDA.R, malonaldehyde in the root.

This heatmap (**Figure 9**) visually summarizes the differential effects of CuO or Fe_2_O_3_ nanoparticle treatments (applied via soil or foliar spray) on various physiological and biochemical parameters in pomegranate plants under both control and nematode-infected conditions. The heatmaps revealed significant differences between the treatments on the left side and the parameters on the top (**Figure 9**). Infected pomegranate plants showed increased stress indicators (MDA.L., MDA.R., and Pro.R.) when exposed to nematodes. Conversely, these conditions negatively impacted the plants’ growth (B.S., L.A.) and photosynthetic pigments (Chl *a*, Chl *b*, and Carot.). When pomegranate plants faced nematode infection, Fe_2_O_3_ NPs (whether sprayed or added to the soil) had moderately positive impacts on several indicators, including MDA.R, Pro.L., Pro.R., and TA.L. The most significant benefit, a highly positive effect, was seen on GSH.L, particularly with the foliar application of Fe_2_O_3_ NPs. Likewise, CuO NP treatments (spray and soil) in nematode-infected plants exhibited moderate positive correlations across three categories: stress indicators (MDA.L, MDA.R, Pro.L, and Pro.R), phytochelatin compounds (TTs.L., TA.L., NPTs, and NPTs.L.), and non-enzymatic antioxidants (T.Ph. and T.Flav.). When Fe_2_O_3_ and CuO NPs were treated alone (by soil and spray), the levels of NPTs and growth characteristics (B.S., L.A., Chl *a*, Chl *b*, and Carot.) were significantly and somewhat higher. However, these treatments showed a comparatively unfavorable connection with the other attributes (**Figure 9**).

## 4 Discussion

Root-knot nematodes, *Meloidogyne* spp., are the most significant category of plant parasitic nematodes found worldwide, albeit more prevalent in warmer climes. To explore the potential nematicidal effects of CuO and Fe_2_O_3_ NPs, this study investigated their impact on pomegranate metabolism. The exact method by which CuO or Fe_2_O_3_ NPs act on nematodes remains incompletely understood. Our research indicates that applying CuO or Fe_2_O_3_ improved pomegranate growth and metabolic activities by reducing the negative impacts of nematode infection. CuO and Fe_2_O_3_ treatments significantly reduced the number of juveniles, egg masses, and root galls, indicating that NP treatments might alter the cellular structure of nematodes. According to Ma et al. (2009), heavy metals in *Caenorhabditis elegans* produce toxicity by rupturing the cell membrane and shifting the cations linked to proteins. The reduction in cellular energy by heavy metals like Cu^2+^ can modify mitochondrial function, raise stress levels by generating ROS, and set off the pathways leading to neuronal cell death (Tauseef et al., 2021). It was discovered that paralyzing animals with Cu^2+^ reduced their ability to forage and reproduce (Karlsson et al., 2013; Tauseef et al., 2021). Furthermore, increased Fe levels have been linked to peroxide sensitivity, oxidative damage, and a shortened lifespan in *Caenorhabditis elegans* (Valentini et al., 2012; Amigoni et al., 2021). As a consequence, the nematodes absorbed more iron at this dose, and it was more bioavailable, indicating a severe toxic effect (Benedetti et al., 2023). Applying nanomaterials can either directly or indirectly decrease plant nematodes by enhancing the production of compounds that protect plants (El-Sherif et al., 2019; e Silva et al., 2025) or by negatively affecting them (Elarabi et al., 2022). According to Khan and Siddiqui (2020), an atypical shape of juveniles could be caused by AgNPs or ZnONPs altering lipids, glycogen, and mucopolysaccharides on the nematode’s hypodermis and cuticle. Rohner et al. (2007) proved that the nanoscale significantly enhanced absorption and, thus, bioavailability *in vivo*.

The number of branches and leaf areas suggested that CuO or Fe_2_O_3_ NP (50 mg/L) treatments might promote pomegranate growth. Nematode infection damages roots by invading cells, forming feeding sites, and disrupting nutrient and water absorption (Willig et al., 2023). Compared to infected plants, CuO and Fe_2_O_3_ treatments increased the number of side branches and leaf areas, suggesting a decrease in nematode infection. Furthermore, CuO or Fe_2_O_3_ treatments significantly improved infected pomegranate growth *via* photosynthetic pigment enhancements. Nematode infection reduced the content of photosynthetic pigments in pomegranate leaves. Additionally, CuO and Fe_2_O_3_ treatments in conjunction with infection significantly decreased the number of juveniles, egg masses, and root galls on infected plants. This led to a significant increase in plant-infected growth parameters.

Our study demonstrated that MDA content was elevated in nematode-infected plants, indicating oxidative stress. This response may involve the generation of ROS (Agrawal et al., 2002). In contrast, nematode-infected plants treated with CuO or Fe_2_O_3_ reduced MDA levels and produced more chlorophyll and carotenoids. Similarly, Faraz et al. (2022) found that *Brassica juncea* grew more when CuO NPs (2-8 mg/L) were sprayed on its leaves. This was because the plants had a better photosynthetic rate and an enhanced antioxidant capacity; higher concentrations, on the other hand, did not affect growth. Also, Fe NP treatments increased non-enzymatic antioxidants, which decreased H_2_O_2_ and MDA production.

Proline plays a crucial role in stress regulation by eliminating free radicals (Smirnoff and Cumbes, 1989). Pomegranate plants infected with nematodes had elevated proline levels, indicating a role in mitigating oxidative stress. Similarly, proline was higher in tomatoes infected with nematodes (El-Zawahry et al., 2021). Conversely, plants treated with CuO and Fe_2_O_3_ showed reduced osmolyte synthesis and lower proline levels, which suggested less stress.

Recently, it has become clear how important sulfur compounds are for plants to resist disease. The majority of plant tissues contain high levels of GSH, which is involved in both the detoxification of ROS and methylglyoxal (Hasanuzzaman et al., 2017). Our findings revealed that GSH concentrations significantly increased in nematode-infected plants compared to healthy plants. Treatment of nematode-infected plants with CuO and Fe_2_O_3_ NPs led to a significant elevation in GSH levels. These findings indicate that NPs boost glutathione levels, enhancing plants’ resistance to nematode infection. According to Hasan et al. (2022), GSH levels were elevated in Arabidopsis col-0 during the migratory stage of the cyst nematode infection. This finding may point to increased gene expression for GSH generation. The observed upregulation of *GSH1* and *GSH2* genes could be attributed to the increased demand for glutathione during the migratory stage of nematode infection. GSH may play a crucial role in restoring redox homeostasis and regulating the biosynthesis of the sulfur-containing phytoalexin camalexin (Siddique et al., 2014).

NPTs are crucial for heavy metal detoxification and can serve as oxidative stress markers (Salbitani et al., 2023). In the current study, the nematode infection resulted in a drop in NPT levels, indicating that oxidative stress had been caused in the infected plants. Because NPTs prevent PT oxidation and maintain protein function and structure, they are more vulnerable to alterations caused by oxidative stress in cells (Yang and Guan, 2015).

CuO and Fe_2_O_3_ treatments on nematode-infected plants increased the content of NPTs. The elevated NPT levels may help plants resist infection stress. These results imply that NPs help plants defend against nematode infection by increasing the number of NPTs. While NPs can increase non-protein thiols (Farghaly et al., 2023), their impact on thiol levels in nematode-infected plants remains unexplored.

In practically every organism, one of the best protein-based defense mechanisms is protein thiols (Meyer et al., 2012). Our findings demonstrate that nematode infection triggers an increase in PT levels in infected plants. This implies that plants fight oxidative stress during nematode attacks using a previously unidentified mechanism. CuO and Fe_2_O_3_ treatments further increased the PT levels in nematode-infected plants, indicating that they actively support ROS detoxification and regulate intracellular oxidative stress. There isn’t any research in the literature to suggest that NPs enhance the synthesis of PTs in plants infected with nematodes.

Antioxidants function to stop ROS generation and alter environmental signals (Foyer and Noctor, 2005). Total antioxidant activity offers valuable insights into a plant’s ability to combat oxidative stress. The antioxidant content of nematode-infected plants was much lower than in healthy plants, suggesting that infection increased ROS production, which stunted plant growth. CuO and Fe_2_O_3_ treatments mitigated nematode-induced reductions in total antioxidants. NPs may enhance plant growth during nematode infection by boosting antioxidant levels.

Phenolic compounds, which serve as antioxidants to eliminate excessive ROS brought on by stress, are some of the most prevalent secondary metabolites in plant life. The results showed that nematode infection significantly increased the content of phenolics in infected plant leaves. This increase suggests that nematode infection triggers a defensive mechanism at the cellular level. Rahaman et al. (2020) propose that phenolics contribute to wheat resistance against *Pratylenchus thornei*. Bhuiyan et al. (2009) found that plant-microbe interactions result in the buildup of lignin-like phenolics or lignin compounds in cell walls. CuO and Fe_2_O_3_ treatments did not affect the phenolic levels in nematode-infected plants, which meant they were still higher than in healthy plants. Further research is needed to determine the precise mechanism by which NPs contribute to the phenolic compounds of nematode-infected plants.

Flavonoids often confer protection against (a)biotic stresses (Shah and Smith, 2020; Mahmoud et al., 2024). Our data showed that nematode infection caused a significant increase in flavonoids in infected plants. The precise processes by which flavonoids reduce worm movement and chemotaxis are still unknown (Shah and Smith, 2020). CuO and Fe_2_O_3_ treatments further increased the production of flavonoids in nematode-infected plants due to their active roles in ROS detoxification. While NPs can elevate flavonoid levels, their impact on nematode-infected plants remains unexplored.

## 5 Conclusion

Root-knot nematodes (*Meloidogyne* spp.) pose a serious threat to pomegranate cultivation, causing severe damage and compromising crop productivity. Nanoparticles (NPs) provide a sustainable alternative to chemical nematicides for nematode control. CuO and Fe_2_O_3_ NPs can be used to regulate *Meloidogyne javanica* in pomegranate farming in a sustainable and environmentally friendly manner. This approach may enhance plant resilience by stimulating the synthesis of antioxidants, flavonoids, thiols, and photosynthesis pigments. Advanced plant protection strategies require a deep understanding of the complex defense mechanisms that NPs activate in plants. Fe_2_O_3_ NPs are beneficial even without nematode infestation, but the combination with nematodes possibly triggers a stronger defense response. CuO NPs reflect a protective role in oxidative stress mitigation.

## Data Availability

The original contributions presented in the study are included in the article/supplementary material. Further inquiries can be directed to the corresponding author.
